# Bacteria-Derived Hemolysis-Related Genes Widely Exist in Scuticociliates

**DOI:** 10.3390/microorganisms8111838

**Published:** 2020-11-22

**Authors:** Jing Zhang, Kai Chen, Chuanqi Jiang, Wentao Yang, Siyu Gu, Guangying Wang, Yishan Lu, Wei Miao, Jie Xiong

**Affiliations:** 1 Shenzhen Institute of Guangdong Ocean University, Shenzhen 518120, China; zhangjing@ihb.ac.cn (J.Z.); jiangchuanqi@ihb.ac.cn (C.J.); fishdis@163.com (Y.L.); 2 Key Laboratory of Aquatic Biodiversity and Conservation, Institute of Hydrobiology, Chinese Academy of Sciences, Wuhan 430072, China; chenkai@ihb.ac.cn (K.C.); yangwentao3000@126.com (W.Y.); gusiyu12@126.com (S.G.); wangguangying@ihb.ac.cn (G.W.); miaowei@ihb.ac.cn (W.M.); 3 Guangdong Provincial Engineering Research Center for Aquatic Animal Health Assessment, Shenzhen 518120, China; 4 Shenzhen Dapeng New District Science and Technology Innovation Service Center, Shenzhen 518119, China; 5 University of Chinese Academy of Sciences, Beijing 100049, China; 6 State Key Laboratory of Freshwater Ecology and Biotechnology of China, Wuhan 430072, China; 7 CAS Center for Excellence in Animal Evolution and Genetics, Kunming 650223, China

**Keywords:** scuticociliatosis, *Uronema marinum*, MAC genome, hemolysis-III like protein

## Abstract

Scuticociliatosis is an invasive external or systemic infection caused by ciliated protozoa, mainly those within the subclass Scuticociliatia (scuticociliates). Many scuticociliates are fish pathogens, including *Miamiensis avidus*, *Philasterides dicentrarchi*, *Pseudocohnilembus persalinus*, and *Uronema marinum*. Our previous study showed that hemolysis-related genes derived from bacteria through horizontal gene transfer (HGT) may contribute to virulence in *P. persalinus*. Hemorrhagic lesions are a common feature of scuticociliatosis, but it is not known whether other scuticociliates also have bacteria-derived hemolysis-related genes. In this study, we constructed a high-quality macronuclear genome of another typical pathogenic scuticociliate, *U. marinum*. A total of 105 HGT genes were identified in this species, of which 35 were homologs of hemolysis-related genes (including hemolysin-like genes) that had previously been identified in *P. persalinus*. Sequencing of an additional five species from four scuticociliate families showed that bacteria-derived hemolysis-related genes (especially hemolysin-like genes) are widely distributed in scuticociliates. Based on these findings, we suggest that hemolysin-like genes may have originated before the divergence of scuticociliates.

## 1. Introduction

Scuticociliatosis is one of the most serious parasitic diseases in fish worldwide, with a high mortality rate in cultured marine fish, e.g., olive flounder and turbot [1,2]. Associated outbreaks of fatal infection have led to serious economic losses. Scuticociliates, which belong to the ciliate subclass Scuticociliatia, have been identified as causative agents of scuticociliatosis. A salient clinical manifestation of scuticociliatosis is hemorrhagic lesions [3]. More than 20 species are facultative parasites that can destroy host tissues, including *Miamiensis avidus*, *Philasterides dicentrarchi*, *Pseudocohnilembus persalinus*, and *Uronema marinum* [4], suggesting a common molecular basis for virulence in scuticociliates. However, many studies on scuticociliates have focused on species identification, morphology, and phylogenetic analysis, and few have examined the molecular basis or mechanism of virulence. One study on *P. dicentrarchi* found that cysteine proteases are activated and secreted into the extracellular medium and probably participate in host tissue invasion and degradation, as well as in the degradation of hemoglobin within erythrocytes [5]. Metalloprotease, phosphatidylcholine (PC)-specific phospholipase D (PC-PLD), and PC-specific phospholipase C (PC-PLC) activities have been detected in *U. marinum*-infected tissues, and may play an important role in host cell penetration [6].

In 2015, we sequenced the first macronuclear (MAC) genome of a scuticociliate, *P. persalinus*. Comparative genomics analysis showed that the MAC genome is relatively small compared with other free-living ciliates and that the protein domain composition resembles that of an obligate fish parasite, *Ichthyophthirius multifiliis*. More importantly, the *P. persalinus* MAC genome harbors many bacteria-derived genes that encode proteins which may be involved in cell adhesion, hemolysis, and heme utilization processes [7].

Similarly to *P. persalinus*, *U. marinum* is a facultative parasite [8] that can be cultured at a high density in a variety of media and cryopreserved in liquid nitrogen using glycerol and fetal bovine serum [9]. To explore the molecular basis of the virulence of scuticociliates, we sequenced the MAC genome of *U. marinum* using Oxford Nanopore Technologies long-read sequencing and acquired a high-quality genome assembly. Horizontal gene transfer (HGT) analysis showed that *U. marinum* has similar hemolysis-related genes to *P. persalinus*. Sequencing of five other scuticociliates (members of four families) using Illumina short-read sequencing showed that hemolysis-related genes are widely distributed in scuticociliates. These findings suggest that these genes originated in an ancestor of the scuticociliates through HGT.

## 2. Methods

### 2.1. Ciliate Culture and Identification

**Figure 1:**
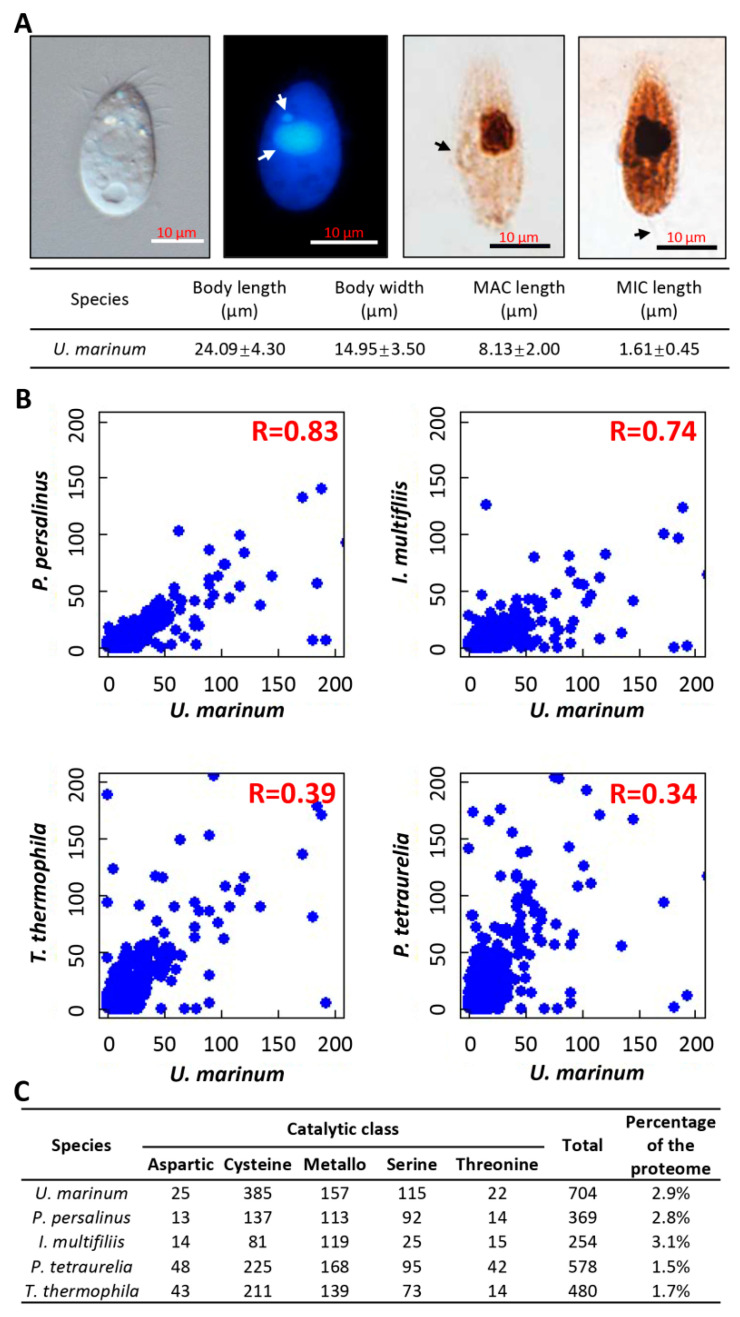
Genomic evidence for parasitism in *U. marinum*. (**A**) *U. marinum* morphology under (left to right) light microscopy, 4′,6-diamidino-2-phenylindole (DAPI) staining, and silver staining, with the latter showing the dorsal and ventral surfaces. White arrows indicate micronucleus (MIC) and macronucleus (MAC); black arrows indicate the oral cilium and the caudal cilium. (**B**) Correlations of protein domain composition between *U. marinum* and four other ciliates. Both the horizontal and vertical axes represent the number of protein domains. (**C**) Number of protease genes in five different ciliates.

*U. marinum* (a gift from Dr Xuming Pan, Harbin Normal University) was isolated from seawater in Qingdao (QD), China (36°08′ N, 120°43′ E; salinity 3.5%) [10]. The body shape was elongated with a rounded posterior and bluntly pointed anterior end. The length and width of cells averaged 24.09 μm and 14.95 μm, respectively. For 4′,6-diamidino-2-phenylindole (DAPI) staining, the cells were fixed by 37% formaldehyde and mounted with DAPI, then incubated for 5 min at 4 °C. A total of 10 μL cell suspension was applied to the slides. Cell staining was observed by means of fluorescence microscopy using an Olympus BX51 (Olympus Corp., Tokyo, Japan). The staining showed two typical types of nucleus: MAC and micronucleus (MIC), of 8.13 μm and 1.61 μm in diameter, respectively. For silver staining, the method is briefly described as follows [11]. The enriched cells were fixed with 50% formalin solution for 5 min at room temperature (RT), washed several times, then added an equal-volume mixture of 10% formalin and Fernandez–Galiano solution. The cells were heated in a 60 °C water bath under continual observation until the color of the cell body became brownish black, and were then added to the same volume of 5% Na_2_S_2_O_3_ solution. Finally, the cells were tipped onto the slides with glass pipette and observed using light microscopy with an Olympus BX51 (Figure 1A).

The *U. marinum* 18S small subunit (SSU) ribosomal DNA (rDNA) gene was PCR-amplified using the universal ciliate primer pairs 18S-F (AACCTGGTTGATCCTGCCAGT) and 18S-R (TGATCCTTCTGCAGGTTCACCTAC) as follows: Denaturation at 94 °C for 5 min; 33 cycles of denaturation at 94 °C for 30 s, annealing at 55 °C for 30 s, and extension at 72 °C for 2 min; and a final extension step at 72 °C for 5 min [12]. The 18S rDNA gene shared 99.11% sequence identity with the previously reported *U. marinum* (GenBank accession: GQ259745.1) [13].

The *U. marinum* cells were cultured in PGY medium (proteose peptone 1.5%, glucose 0.5%, yeast exact 0.5%, pH 8.0) containing sterilized seawater (filtered through a 200 μm pore membrane) at 25 °C. Bacterial contaminations were reduced by adding 200 μg/mL normocin (Invivogen, San Diego, CA, USA) to the culture medium.

Five other scuticociliates were also isolated. One was isolated from seawater in Qingdao (QD), China; two were isolated from a fish farm (*Larimichthys polyactis*) in Ningde (ND), China; and two were isolated from seawater in Shenzhen (SZ) Bay Port, China. All five scuticociliates were cultured in the medium described above supplemented with wheat (one grain per 10 mL sterilized water). Bacterial contaminations were reduced using the method above. DNA was extracted using the phenol/chloroform method. PCR amplification for 18S rDNA was conducted using the primers as before. Species identification was based on Basic Local Alignment Search Tool (BLAST) search results against the GenBank database (Table S1).

### 2.2. DNA and RNA Sample Preparation and Sequencing

*U. marinum* cells were lysed in TEN buffer (100 mM Tris-HCl at pH 8.0, 50 mM EDTANa_2_ at pH 8.0, 1 M NaCl) containing 0.1% β-mercaptoethanol, 30 mg proteinase K, and 2.5% SDS. DNA was then extracted from a chloroform:isoamyl alcohol (24:1) mixture and precipitated in isopropanol. The DNA pellet was washed in 70% ethanol and resuspended in sterile water. The contimated RNA was removed by incubation at 37 °C with 100 µg/mL RNase A for 45 min. A Blood and Cell Culture DNA Midi Kit (Qiagen Sciences, Germantown, MD, USA) was used to purify DNA according to the manufacturer’s protocols. DNA was then purified using an equal volume of AMPure XP beads (Beckman Coulter, Indianapolis, IN, USA). DNA quality and concentration were checked using a Qubit 3.0 Fluorometer and 0.8% agarose gel electrophoresis. Total DNA from *U. marinum* was sequenced using Oxford Nanopore sequencing (for long reads) and MGI sequencing (for short reads). For Nanopore sequencing, a library was prepared using 1D Genomic DNA sequencing (SQK-LSK109 Ligation Sequencing Kit, Oxford Nanopore Technologies) according to the manufacturer’s protocols. An average fragment length of 8 Kb was selected with a high-throughput automated DNA (fragment) recovery system (Sage Science, Beverly, MA, USA) and was used to construct the library. The final library was loaded onto a PromethION flow cell and monitored using MinKNOW software (version 1.15.1) over a 24-h sequencing period. For MGI sequencing, a library was prepared using a DNA Sample Preparation Kit, as recommended by the manufacturer. The library was used for 300 bp paired-end sequencing using a MGISEQ-2000 sequencer (MGI Tech Co., Ltd., Shenzhen, China).

Total RNA was extracted from *U. marinum* cells in the growth phase using an RNeasy Protect Cell Mini Kit (Qiagen, Valencia, CA, USA), as described in the *Tetrahymena* Functional Genomics Database (TetraFGD) [14]. Pair-end (150 bp) Illumina sequencing libraries were constructed according to the manufacturer’s protocols and analyzed with a HiSeq 4000 sequencer (Illumina, San Diego, CA, USA). Raw read adaptors were trimmed with Trim-Galore version 0.4.0 [15] and mapped to the *U. marinum* genome using TopHat version 2.0.9 [16].

Total DNA was extracted from the other five scuticociliates using a method previously described for *Tetrahymena* [17]. Briefly, cells were harvested by centrifugation at 5000 *g* for 3 min after filtering with a 200 µm pore membrane (excluding the bacterial aggregates), and then lysed with urea buffer (20 mM Tris-HCl pH 7.4, 50 mM NaCl, 12.5 mM EDTANa_2_ pH 8.0, 2% SDS, 42% urea). DNA was extracted using a phenol/chloroform/isoamyl alcohol (25:24:1) mixture and precipitated in isopropanol. The DNA pellet was washed in 70% ethanol and resuspended in sterile water. DNA samples were sequenced using the NovaSeq 6000 platform (Illumina, San Diego, CA, USA) in 150 bp × 2 mode according to the manufacturer’s protocols. The quality of raw sequence reads was assessed using FastQC version 0.11.8 and Trim-Galore [15,17]. After adaptor trimming and filtering low-quality reads, the clean sequence data were used for further bioinformatics analysis.

### 2.3. U. marinum Genome Assembly and Gene Prediction

Error-prone nanopore long reads were used for de novo assembly of the *U. marinum* genome using Canu version 1.2 [18]. The higher-accuracy MGIseq short reads were used to correct errors in long read assembly (i.e., polishing). In detail, MGIseq paired-end reads were mapped to the long-read assembly using BWA-MEM and the assembly was polished using Pilon version 1.2.3 [19,20]. A total of three rounds of polishing were performed, in accordance with Canu recommendations [18].

For gene prediction, RNA-Seq data were de novo assembled using Trinity and reference-guided assembled using the TopHat and Cufflinks pipeline [16,21]. A combination of de novo and reference-guided assembled transcripts were validated by aligning putative transcripts to the assembled genome using PASA [22]. The full-length transcripts determined by PASA were used to train the Augustus and GlimmerHMM gene prediction software [23]. The training parameters were then used by the two programs for de novo prediction of gene models. As Augustus software can accept cDNA or protein evidence, assembled transcripts were also used as cDNA evidence for Augustus. Finally, an integrated set of gene models was created using Evidence Modeler by merging all of the predicted gene models [24]. Protein domains encoded by the genes were annotated using InterProScan [25]. Genes from *U. marinum*, *P. persalinus*, and *I. multifliis* were taken to perform all-by-all comparisons among ciliates using BLASTP with an E-value less than 1 × 10^−5^. Ortholog groups (clusters) were then annotated using OrthoMCL, which provided the best overall balance of sensitivity and specificity for multiple species ortholog clustering [26]. The important parameter inflation index of OrthoMCL was set at 1.5 to balance sensitivity and selectivity, as used in OrthoMCL-DB construction.

### 2.4. Identification of HGT Genes in U. marinum

HGT genes were identified using a published pipeline [7]. In detail, all predicted genes were BLASTP searched against the NCBI non-redundant protein database using an E-value threshold of 1 × 10^−5^, and any prokaryote genes that had a best hit were regarded as candidate HGT genes.

Second, phylogenetic approaches were used to validate the HGT genes identified in *U. marinum*. Briefly, we divided the NCBI non-redundant database into a eukaryote database and a prokaryote database using blastdbcmd [27]. Next, all candidate genes retrieved from the first step were independently BLASTP searched against the two databases in order to retrieve both eukaryotic and prokaryotic homologs. *U. marinum* proteins with more than five homologs with an E-value of less than 1 × 10^−5^ in prokaryotes but no homologs with an E-value of less than 1 × 10^−5^ in eukaryotes were not included in the phylogenetic trees because of their low similarity to eukaryotic proteins. Such proteins were defined as HGT proteins if the E-value of the best prokaryote hit divided by the E-value of best eukaryote hit was less than 1 × 10^−5^. For homologs with an E-value of less than 1 × 10^−5^ in both prokaryotes and eukaryotes, a phylogenetic tree was used to determine whether the gene originated from prokaryotes or eukaryotes. Two programs (FASTTREE and PHYML) were used to construct the phylogenetic tree [28,29]. All homologs with an E-value of less than 1 × 10^−5^ were used to construct the phylogenetic tree in FASTTREE due to its fast computation speed. In PHYML, only the top 10 homologs (if present) were used to construct the phylogenetic tree. Sequences were aligned in MUSCLE before constructing the phylogenetic tree. A gene was accepted as being encoded by an HGT gene originating in prokaryotes if it clustered within a prokaryotic clade that had a eukaryotic outgroup.

### 2.5. Homologs of U. marinum HGT Genes in Other Scuticociliates

In addition to *U. marinum*, we sequenced the genome/transcriptome of the other five scuticociliates. For four species (*Uronemita* sp. ND, *Uronema* sp. ND, *Parauronema* sp. SZ, and *Uronema* sp. SZ), we generated only short reads (Illumina) to assemble the draft genomes using Megahit [30]. For one species (*Paralembus* sp. QD), the transcriptome was sequenced and assembled using Trinity [31]. The removal of contaminating bacterial genes was based on a BLASTX search against the NCBI non-redundant protein database and the guanine-cytosine (GC) distribution (as for *U. marinum*). Regions homologous to *U. marinum* and *P. persalinus* HGT genes in the five scuticociliates were identified by TBLASTN searches, and gene models in these homologous regions were predicted using Augustus and GeneWise [32,33,34]. All the raw sequenced data were deposited in the Genome Sequence Archive (GSA) database under the BioProject number PRJCA003485, and all the assembled sequences and annotation were deposited in the Genome Warehouse (GWH) under the submission number WGS012693.

## 3. Results and Discussion

### 3.1. The U. marinum MAC Genome

Polyploidy is a common feature of ciliate MACs. For example, the MAC ploidy is ~45 C in *Tetrahymena*, ~800 C in *Paramecium*, and more than 1000 C in *Oxytricha* [35,36,37]. Therefore, as with those species, bulk DNA from *U. marinum* should also predominantly comprise MAC DNA. A preliminary MAC genome of 91.7 Mb was assembled using long reads generated by nanopore sequencing. The GC content distribution of the preliminary assembly had two peaks at 0.18 and 0.64 (Figure S1). Sequences with a high GC content were found to be a result of bacterial contamination. Based on the GC distribution and a BLAST search (the same procedure applied for *P. persalinus*), bacterial contaminations were successfully removed, and 86.8 Mb MAC genome assembly with 403 scaffolds was obtained for *U. marinum*. In all, 54% (218 out of 403) of scaffolds were chromosome-level assemblies with telomere sequence repeats ([C4A2]_n_) at both ends, indicating that the *U. marinum* MAC genome assembly was of high quality compared with other ciliates (Table 1) [7,38,39,40]. The *U. marinum* genome was 60% larger than that of a previously sequenced facultative scuticociliate parasite, *P. persalinus*, and 80% larger than that of the obligate fish parasite *I. multifiliis*, but 20% smaller than that of the free-living ciliate *Tetrahymena thermophila*.

Using both de novo and homology-based gene prediction pipelines, a total of 24,582 genes were identified in *U. marinum* (Table 1). This number was about 1.86-fold greater than that of *P. persalinus* and comparable to that of *T. thermophila*. Segmental DNA duplications were found to be the main contributors to the high gene number in *U. marinum* (Table S2). *U. marinum* shared many orthologs with *P. persalinus* (*n* = 3235) that were absent in *I. multifiliis* (Figure S2). Global protein domain analysis showed that the domain composition of *U. marinum* predicted proteins was similar to that of *P. persalinus* and *I. multifiliis*, but different from that of *T. thermophila* (Figure 1B). More importantly, we found that *U. marinum*, *P. persalinus*, and *I. multifiliis* had similar proportions of parasitic lifestyle-related gene families—they all contained a higher percentage of proteases compared with free-living ciliates (Figure 1C). Both *U. marinum* and *P. persalinus* had more proteases than *I. multifiliis*, especially in the cysteine and serine catalytic classes (Figure 1C), which may contribute to virulence in scuticociliates.

### 3.2. U. marinum Acquired Hemolysis-Related Genes through HGT

**Figure 2:**
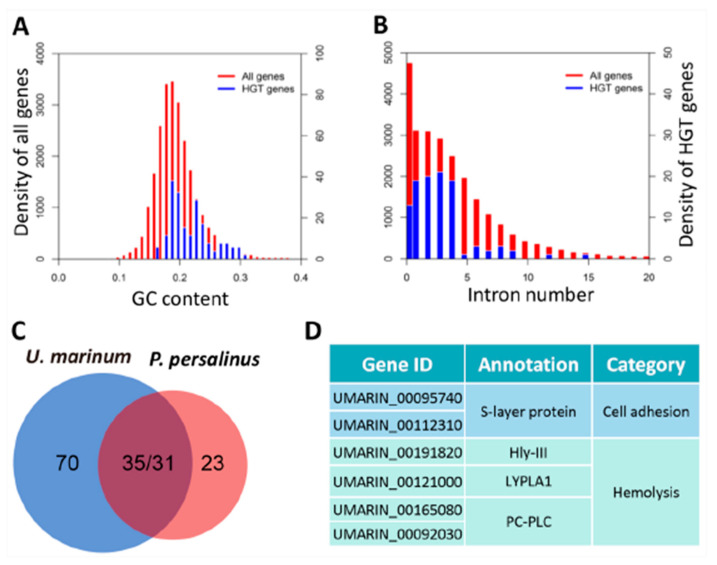
Horizontal gene transfer (HGT) genes in *U. marinum*. (**A**) Guanine-cytosine (GC) content of the 105 HGT genes. (**B**) Distribution of intron number in the 105 HGT genes. (**C**) Venn diagram showing the numbers of specific and homologous HGT genes in *U. marinum* and *P. persalinus*. (**D**) Potential hemolysis-related HGT genes identified in *U. marinum*.

MAC genome sequencing showed that the facultative parasite *P. persalinus* acquired many genes from bacteria through HGT, especially hemolysis-related genes [7]. The histophagous ciliate *U. marinum* is closely related to *P. persalinus* and can also induce systemic scuticociliatosis in marine fish [41,42]. To determine whether *U. marinum* also acquires bacterial genes through a similar mechanism, we identified all HGT genes in *U. marinum*. A total of 105 putative HGT genes were identified in *U. marinum*. The GC content of these genes was found to be similar to the other *U. marinum* genes (Figure 2A), and 87.62% were predicted to have introns, a typical feature of eukaryotic genes (Figure 2B). These results suggest that the 105 genes were not derived from bacterial contamination.

Comparison of HGT genes between *U. marinum* (105 genes; Table S3) and *P. persalinus* (54 genes) showed that 33.3% (*n* = 35) of *U. marinum* HGT genes are homologs of *P. persalinus* genes (Figure 2C), including hemolysis-related genes, such as those encoding the cell adhesion proteins hemolysin III-like (Hly-III-like), lysophospholipase L1 (LYPLA1), and PC-PLC (Figure 2D).

Cell adhesion is an initial process in the infection response involving the recognition of signals between the pathogen and the host [43]. Different sets of cell adhesion genes were identified in the HGT genes of both *U. marinum* and *P. persalinus*. In *P. persalinus*, two Ig family genes encode an He_PIG domain (PF05345) that contains a conserved core region of about 90 residue repeats found in several haemagglutinins and other cell-surface proteins, indicating that these proteins may contribute to cell adhesion [7]. In *U. marinum*, cell adhesion gene (UMARIN_00095740 and UMARIN_00112310)-encoded proteins include a surface layer (S-layer) family protein. S-layers are the outermost proteinaceous cell envelope structures, found on members of nearly all taxonomic groups of bacteria and archaea [44]. The function of cell adhesion is very different between species, which may be related to species-specific cell recognition during infection.

LYPLA1, PC-PLC, and Hly-III-like proteins are reported to contribute to hemolysis. Two *lypla1* genes (UMARIN_00121000 and UMARIN_00165080) and one *pc-plc* gene (UMARIN_00092030) were identified as HGT genes in *U. marinum*. LYPLA1 and PC-PLC are phospholipases that can destroy the cell membrane and function as virulence factors in the bacterial invasion of host cells [45]. Destruction of the host surface cells is necessary for pathogens to gain access to nutrients such as hemocytes [46,47].

**Figure 3:**
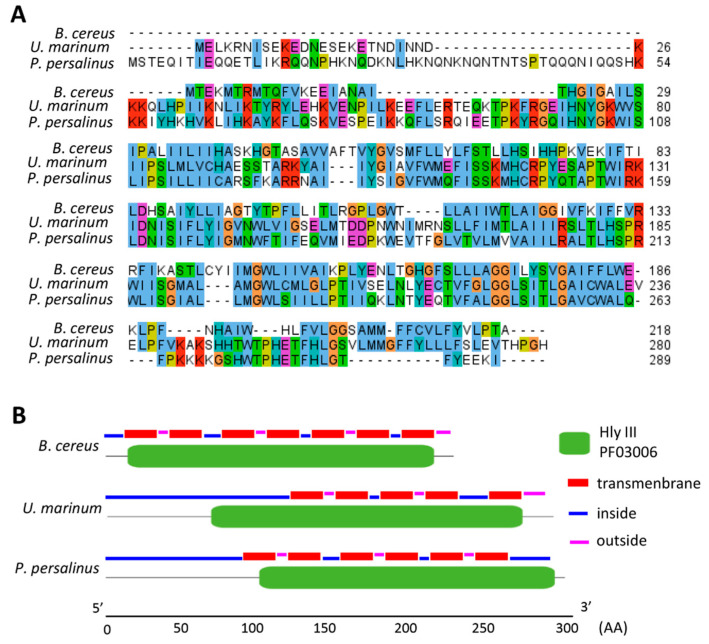
Conservation of *hly-iii* genes. (**A**) Sequence alignment of predicted Hly-III-like proteins in *U. marinum*, *P. persalinus*, and *B. cereus*. Color scheme was based on the default settings on the website http://ekhidna.biocenter.helsinki.fi/pfamz/clustal_colours. (**B**) Conserved domain predicated through Pfam database and transmembrane predicated by TMHMM (http://w3ww.cbs.dtu.dk/services/TMHMM/).

Hly-III is hemolytic toxin found in *Bacillus cereus* that plays an important role in destroying blood cells [48]. The toxin acts by forming an oligomeric pore in three steps: The protein first binds to the erythrocyte surface, and then monomers assemble to form the transmembrane pore, leading to cell lysis. A Hly-III-like protein encoded by the HGT gene (UMARIN_00191820) identified in *U. marinum* showed high-sequence similarity to the Hly-III-like protein previously identified in *P. persalinus*, and shared a conserved domain (Pfam accession: PF03006) and several transmembrane helices with *B. cereus* Hly-III (Figure 3A,B). These results indicate that the Hly-III-like protein is a key protein involved in forming hemorrhagic lesions in scuticociliatosis.

### 3.3. Hemolysis-Related Genes Are Widely Distributed in Scuticociliates

**Figure 4:**
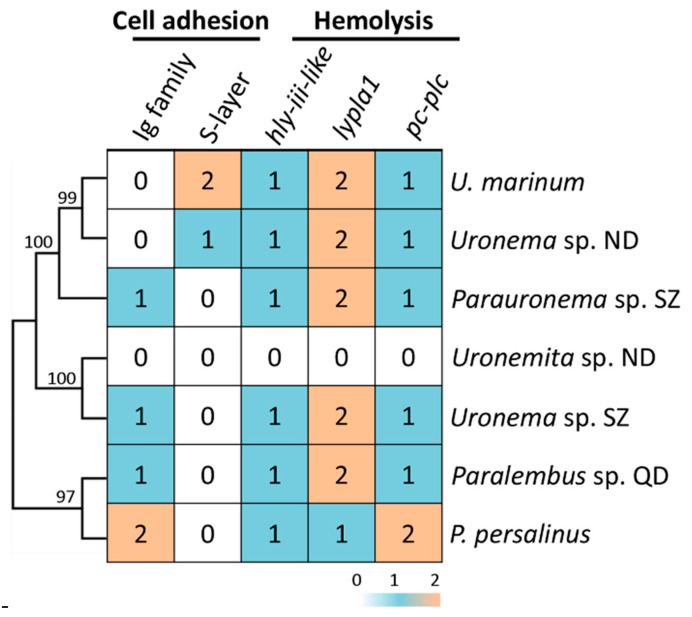
Distribution of hemolysis-related genes in seven scuticociliates. Numbers indicate the number of homologs identified in each species. The functional category was assigned as described in our previous study [7].

**Figure 5:**
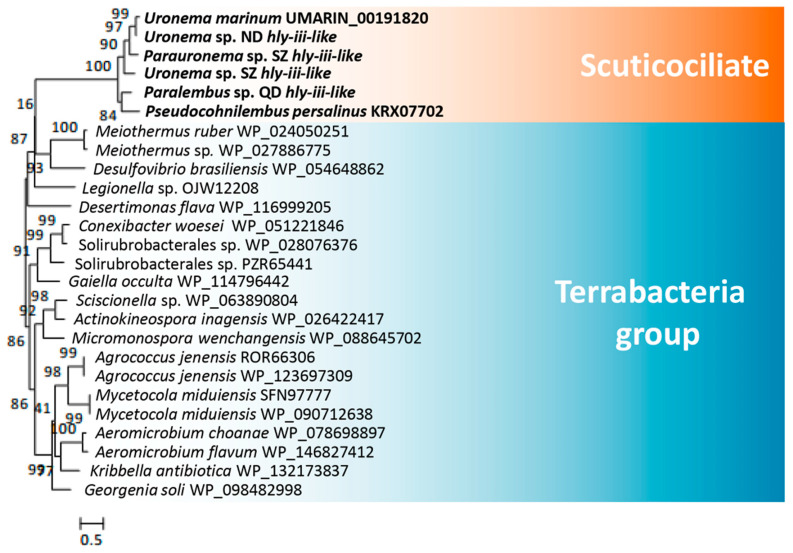
Phylogenetic tree of *hly-iii-like* genes in scuticociliates and bacteria. *hly-iii-like* genes were identified in all scuticociliates. The top 20 prokaryotic BLASTP hits (using the encoded *U. marinum* Hly-III-like proteins as the seed sequence) in the NCBI non-redundant protein database were used to construct the phylogenetic tree.

**Figure 6:**
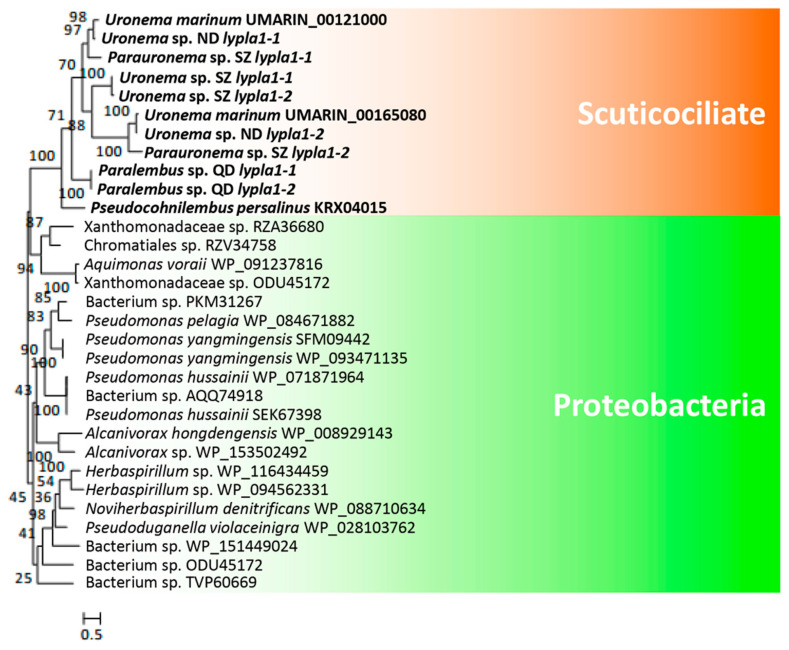
Phylogenetic tree of *lypla1* genes in scuticociliates and bacteria. *lypla1* genes were identified in all scuticociliates. The top 20 prokaryotic BLASTP hits (using the encoded *U. marinum* LYPLA1 proteins as the seed sequence) in the NCBI non-redundant protein database were used to construct the phylogenetic tree.

**Figure 7:**
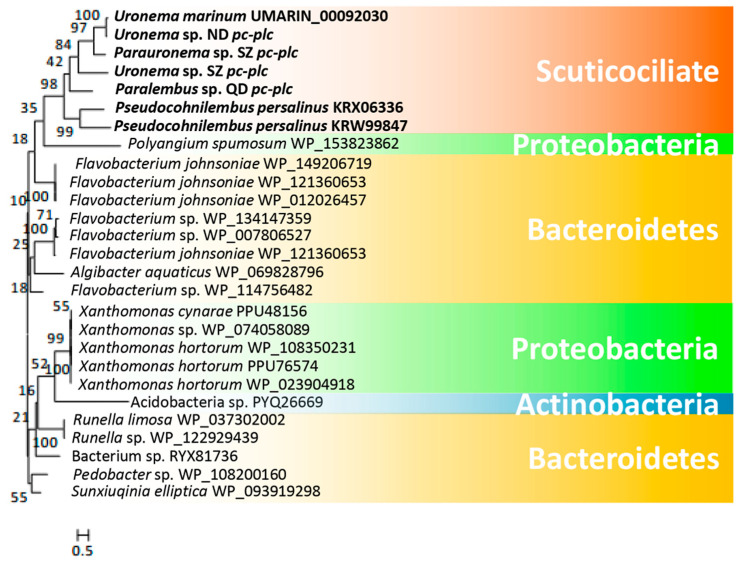
Phylogenetic tree of *pc-plc* genes in scuticociliates and bacteria. *pc-plc* genes were identified in all scuticociliates. The top 20 prokaryotic BLASTP hits (using the encoded *U. marinum* PC-PLC proteins as the seed sequence) in the NCBI non-redundant protein database were used to construct the phylogenetic tree.

*U. marinum* and *P. persalinus* belong to two different families of scuticociliates—Uronematidae and Pseudocohnilembidae. The presence of hemolysis-related HGT genes led us to speculate that these genes originated very early in the evolution of scuticociliates and may be widely distributed in this subclass. To address this possibility, we checked the homologs of hemolysis-related HGT genes in five additional scuticociliates belonging to four families in the class Scuticociliatia—a *Paralembus* sp. isolated in Jiaozhou Bay, a *Uronemita* sp. and a *Uronema* sp. isolated in an *L. polyactis* farm in ND (Fujian province, China), and a *Parauronema* sp. and a *Uronema* sp. isolated in SZ Bay Port, China. Comparative genomics analysis showed that all seven scuticociliates contain homologs of different cell adhesion genes, either Ig family genes or S-layer genes (Figure 4). These results indicate that different scuticociliates have acquired different cell adhesion genes during their evolution. Interestingly, homologs of genes encoding *hly-iii-like*, *lypla1*, and *pc-plc* were found in six of the seven scuticociliates (Figure 4), suggesting a wide distribution of hemolysis genes in scuticociliates. Phylogenetic analysis of *hly-iii-like*, *lypla1*, and *pc-plc* genes showed that these scuticociliate genes cluster into a monophyletic group (Figure 5, Figure 6 and Figure 7) that is separate from bacterial genes. This result suggests that ancestral *hly-iii-like*, *lypla1*, and *pc-plc* genes originated through HGT before the divergence of scuticociliates. Phylogenetic analysis showed that the closest bacterial relatives of *hly-iii-like* are in the Terrabacteria group (Figure 5), whereas the closest bacterial relatives of *lypla1* and *pc-plc* genes are proteobacteria (Figure 6 and Figure 7). These results indicate *hly-iii-like*, *lypla1*, and *pc-plc* genes probably originated from different HGT events involving different bacteria, but more evidence is needed.

## 4. Conclusions

We sequenced and assembled a high-quality MAC genome of *U. marinum*, a typical scuticociliatosis pathogen. As in *P. persalinus*, bacteria-derived hemolysis genes have also been acquired by *U. marinum*. These genes were found to be widely distributed in scuticociliates, suggesting that they have important roles in scuticociliatosis.

## Figures and Tables

**Table 1:** Statistics of the assembled macronuclear (MAC) genomes for five ciliates.

Species	*U. marinum*	*P. persalinus*	*T. thermophila*	*I. multifiliis*	*P. tetraurelia*
Subclass	Scuticociliatia	Scuticociliatia	Oligohymenophorea	Oligohymenophorea	Oligohymenophorea
Genome size (Mb)	86.8	55.5	103.0	47.8	72.1
N50 (Kb)	470	368	521	66	413
Scaffold number	403	288	1148	1375	697
Longest scaffold (Mb)	2.48	2.0	2.2	0.4	1.0
Sequencing method/platform	Nanopore/MGI	Illumina	Sanger	Sanger/454	Sanger
Average guanine-cytosine (GC) content	18%	19%	22%	16%	28%
Assembled chromosome number	218 (54%)	0 (0%)	129 (11.2%)	0 (0%)	8(1%)
Completeness	84.8%	79.5%	85.1%	67.0%	85.5%
Gene number	24,582	13,186	26,460	8062	39,642

## Supplementary Materials

The following are available online at https://www.mdpi.com/2076-2607/8/11/1838/s1, Figure S1. GC distribution of preliminarily assembled genome. Figure S2. Venn diagram showing specific and orthologous genes in *U. marinum*, *P. persalinus*, and *I. multifiliis*. Table S1. Sampling information and species identification for the five scuticociliates. Table S2. Number and type of segmental duplications in *U. marinum* and *P. persalinus*. WGD, whole-genome duplication. Table S3. Description of the 105 HGT genes in the *U. marinum* MAC genome.
